# A multicenter randomized trial of personalized acupuncture, fixed acupuncture, letrozole, and placebo letrozole on live birth in infertile women with polycystic ovary syndrome

**DOI:** 10.1186/s13063-020-4154-1

**Published:** 2020-03-04

**Authors:** Shiya Huang, Min Hu, Ernest Hung Yu Ng, Elisabet Stener-Victorin, Yanhua Zheng, Qidan Wen, Cong Wang, Maohua Lai, Juan Li, Xingcheng Gao, Xinhua Wang, Zhenxing Hu, Tian Xia, Rongkui Hu, Jianping Liu, Xiaohui Wen, Shuna Li, Kewei Quan, Xingyan Liang, Hongcai Shang, Hongxia Ma, Jie Qiao

**Affiliations:** 1 Department of Traditional Chinese Medicine, First Affiliated Hospital of Guangzhou Medical University, Guangzhou, Guangdong China; 2 Institute of Integration of Traditional Chinese Medicine and Western Medicine, Guangzhou Medical University, Guangzhou, Guangdong China; 3 Department of Obstetrics and Gynecology, University of Hong Kong, Hong Kong, Special Administrative Region China; 4 Department of Physiology and Pharmacology, Karolinska Institutet, Stockholm, Sweden; 5 Department of Traditional Chinese Medicine, Second Affiliated Hospital of Guangzhou Medical University, Guangzhou, Guangdong China; 6 Department of Traditional Therapy, Guangdong Provincial Hospital of Traditional Chinese Medicine, Guangzhou, Guangdong China; 7 Department of Urology, First Affiliated Hospital of Guangzhou Medical University, Guangzhou, Guangdong China; 8 Department of Gynecology, Xuzhou Maternity and Child Health Care Hospital, Xuzhou, Jiangsu China; 9 Center for Reproductive Medicine, First Teaching Hospital of Tianjin University of Traditional Chinese Medicine, Tianjin, China; 10 Department of Reproductive Medicine, Affiliated Hospital of Nanjing University of Chinese Medicine, Nanjing, Jiangsu China; 11 Centre for Evidence Based Chinese Medicine, Beijing University of Chinese Medicine, Beijing, China; 12 Department of Obstetrics and Gynecology, Dongguan Hospital of Traditional Chinese Medicine, Dongguan, Guangdong China; 13 Key Laboratory of Chinese Internal Medicine of Ministry of Education and Beijing, Dongzhimen Hospital, Beijing University of Chinese Medicine, Beijing, China; 14 Department of Obstetrics and Gynecology, Third Hospital, Peking University, Beijing, China

**Keywords:** Acupuncture, Polycystic ovary syndrome, Personalized protocol, Fixed protocol, Letrozole

## Abstract

**Background:**

Traditional Chinese medicine (TCM) usually involves syndrome differentiation and treatment. Acupuncture, one form of TCM, requires the selection of appropriate acupoints and needling techniques, but many clinical trials on acupuncture have used fixed acupuncture protocols without accounting for individual patient differences. We have designed a multicenter randomized controlled trial (RCT) to evaluate whether personalized or fixed acupuncture increases the likelihood of live births in infertile women with polycystic ovary syndrome (PCOS) compared with letrozole or placebo letrozole. We hypothesize that letrozole is more effective than personalized acupuncture, which in turn is more effective than fixed acupuncture, and that placebo letrozole is the least effective intervention. Moreover, we hypothesize that personalized acupuncture is more likely to reduce the miscarriage rate and the risk of pregnancy complications compared with letrozole.

**Methods/design:**

The study is designed as an assessor-blinded RCT. A total of 1100 infertile women with PCOS will be recruited from 28 hospitals and randomly allocated to 4 groups: personalized acupuncture, fixed acupuncture, letrozole, or placebo letrozole. They will receive treatment for 16 weeks, and the primary outcome is live birth. Secondary outcomes include ovulation rate, conception rate, pregnancy rate, pregnancy loss rate, changes in hormonal and metabolic parameters, and changes in quality of life scores. Adverse events will be recorded throughout the trial. All statistical analyses will be performed using IBM SPSS Statistics version 21.0 software (IBM Corp., Armonk, NY, USA), and a *P* value < 0.05 will be considered statistically significant.

**Discussion:**

This study will be the first multicenter RCT to compare the effect of personalized or fixed acupuncture with letrozole or placebo letrozole on live birth in infertile women with PCOS. The findings will inform whether personalized acupuncture therapy can be considered an alternative treatment to improve the live birth rate in infertile women with PCOS.

**Trial registration:**

ClinicalTrials.gov, NCT03625531. Registered on July 13, 2018.

Chinese Clinical Trial Registry, ChiCTR1800017304. Registered on July 23, 2018.

## Background

Polycystic ovary syndrome (PCOS) is associated with anovulation; infertility; and pregnancy complications such as pregnancy-induced hypertension, preeclampsia, gestational diabetes, and premature delivery [1, 2]. There are several options for managing anovulatory infertility in women with PCOS, including lifestyle modifications, pharmacological treatment (letrozole, clomid, metformin, and gonadotropins), laparoscopic ovarian diathermy, and in vitro fertilization [3]. Letrozole, an aromatase inhibitor, is the first-line therapy for infertility in women with PCOS [4]. However, letrozole has not been shown to reduce pregnancy complications in infertile women with PCOS [5, 6]. Thus, it is necessary to find an approach to improve the live birth rate and reduce pregnancy complications.

Acupuncture has been used in China for more than 3000 years and is a core component of traditional Chinese medicine (TCM). Jedel et al. [7] reported that acupuncture with manual stimulation and low-frequency electrical stimulation was superior to exercise and to no treatment for improving menstrual frequency and reducing total testosterone levels. Johansson et al. [8] demonstrated that repeated acupuncture resulted in higher ovulation frequency during the treatment period in women with PCOS. In addition, acupuncture has been shown to restore dysfunctional glucose homeostasis as well as adipose tissue gene expression and DNA methylation in patients with PCOS, in part via activation of the autonomic nervous system [9]. Despite these previous findings, a recent large multicenter trial in China demonstrated that acupuncture given as a fixed protocol did not improve the live birth rate in women with PCOS when compared with placebo acupuncture or clomiphene citrate [10]. Pastore et al. claimed that placebo acupuncture showed a similar improvement in the luteinizing hormone (LH) to follicle-stimulating hormone (FSH) ratio as fixed acupuncture in women with PCOS [11]. Due to there being no studies using placebo or no intervention as a comparator to determine the effect of acupuncture on live birth rate, the evidence of acupuncture treatment for infertile women with PCOS is still insufficient.

Acupuncture is a complex treatment in TCM and is tailored to individual patients. According to TCM, the signs and symptoms of each patient should be considered in a differential symptom-based diagnosis before selecting the acupoints. Although several studies have concluded that acupuncture is effective for some disease conditions [12, 13], other studies have found no differences between personalized, fixed, and sham acupuncture on systolic or diastolic blood pressure [14]. Also, a recent study demonstrated no improvement in live birth rate using a fixed acupuncture protocol and following Western medical acupuncture theories [10]. The acupuncture points were selected according to innervation of the ovaries and uterus and differed from TCM theory based on syndrome differentiation and treatment. Thus, it is not certain if acupuncture is more effective than no intervention for ovulation induction and improving live birth rate.

Although no study has shown the efficacy of acupuncture in reducing pregnancy complications, the advantage of acupuncture in losing weight [15], reducing total testosterone levels [7], and improving insulin resistance [16] might provide potential benefits in reducing pregnancy complications such as miscarriage, gestational diabetes, and gestational hypertension.

Although most studies on acupuncture have used a fixed acupuncture protocol, some have demonstrated the efficacy of personalized acupuncture based on disease differentiation and TCM theory [12, 13, 17–19]. However, no studies have investigated acupuncture in the management of anovulatory infertility in women with PCOS. Therefore, we aim to compare the efficacy of personalized acupuncture based on TCM theory, fixed acupuncture, letrozole, and placebo letrozole on the live birth rate in infertile women with PCOS.

## Objectives

The objectives of the present trial are to test the following four hypotheses:

1. Letrozole is more likely than personalized acupuncture to induce ovulation and to result in live birth.
2. Personalized acupuncture is more likely than fixed acupuncture to induce ovulation and to result in live birth.
3. Fixed acupuncture is more likely than placebo letrozole to induce ovulation and to result in live birth.
4. Personalized acupuncture is more likely than letrozole, fixed acupuncture, and placebo letrozole to reduce the miscarriage rate and the risk of pregnancy complications.

## Methods/design

### Study design

This multicenter randomized trial involves four treatment groups. The research protocol is compliant with the Consolidated Standards of Reporting Trials (CONSORT) [20] guidelines and the Standards for Reporting Interventions in Clinical Trials of Acupuncture (STRICTA) [21], as well as with the Standard Protocol Items: Recommendations for Interventional Trials (SPIRIT) statement [22]. The SPIRIT checklist is presented in Supplementary file 1.

### Recruitment

A total of 1100 infertile women with PCOS will be recruited from 28 hospitals in China and will be randomly allocated to the following four groups: personalized acupuncture, fixed acupuncture, letrozole, or placebo letrozole. The women will be recruited through advertisements in local newspapers and bulletin boards in each trial center. Prior to participation, eligible women will sign the consent form after a detailed explanation of the study design and comprehensive counseling.

Inclusion criteria are as follows:

1. Age between 20 and 40 years
2. Chronic oligomenorrhea or amenorrhea. *Oligomenorrhea* is defined as an intermenstrual interval > 35 days or < 8 menstrual bleedings in the past year. Amenorrhea is defined as an intermenstrual interval > 90 days.
3. Hyperandrogenism (either hirsutism or hyperandrogenemia) or polycystic ovaries on ultrasound. Hirsutism is determined by a modified Ferriman-Gallwey (FG) score ≥ 5 at screening examination [23], and biochemical hyperandrogenism is defined as total testosterone (T) > 2.6 nmol/L and free T ≥ 6.0 pg/ml [24]. (The cutoff level might differ for each study site.) Polycystic ovaries are considered present when the number of small antral follicles (2–9 mm in diameter) is ≥ 12 or the ovarian volume is > 10 ml on transvaginal ultrasound [25].
4. At least one patent tube shown by hysterosalpingogram, contrast sonography, or diagnostic laparoscopy within 3 years if the patient does not have a history of abortion or pelvic operation. If the patient has a history of pregnancy and no history of pelvic operation within the past 5 years, she will not be required to undergo a tubal patency test.
5. Sperm concentration ≥ 15 × 10^6^/ml and total motility ≥ 40% or a total motile sperm count ≥ 9 million in the semen analysis of the husband [26].
6. The couple agrees to have regular intercourse (i.e., two or three times per week) during the study period.

Exclusion criteria are as follows:

1. Other endocrine disorders:
  - Patients with hyperprolactinemia (defined as two prolactin [PRL] levels ≥ 25 ng/ml at least one week apart)
  - Patients with FSH levels > 15 mIU/ml. A normal level within the last year is adequate for participation.
  - Patients with uncorrected thyroid disease (defined as thyroid-stimulating hormone [TSH] < 0.2 mIU/ml or > 5.5 mIU/ml). A normal level within the last year is adequate for participation.
  - Patients diagnosed with poorly controlled type 1 or type 2 diabetes mellitus (defined as a glycated hemoglobin [HbA1c] level > 7.0%) or patients receiving antidiabetic medications such as metformin, insulin, thiazolidinediones, acarbose, or sulfonylureas
  - Patients with suspected Cushing syndrome
2. Use of other TCM treatments, including Chinese herbal prescriptions or acupuncture in the past 3 months
3. Use of other Western medications known to affect reproductive function or metabolism in the past 2 months
4. Pregnancy within the past 6 weeks
5. Within 6 weeks postabortion or postpartum
6. Breastfeeding within the last 6 months
7. Not willing to give written consent to the study
8. Additional exclusion criteria:
  - Patients taking other medications known to affect reproductive function or metabolism. These medications include oral contraceptives, depot progestins, hormonal implants (including Implanon; Merck & Co., Whitehouse Station, NJ, USA), gonadotropin-releasing hormone agonists and antagonists, antiandrogens, gonadotropins, antiobesity drugs, antidiabetic drugs such as metformin and thiazolidinediones, somatostatin, diazoxide, angiotensin-converting enzyme inhibitors, and calcium channel blockers. The washout period for these medications will be 2 months, but longer washouts might be necessary for certain depot contraceptive forms or implants, especially when the implants are still in place. A 1-month washout will be required for patients receiving depot progestins.
  - Patients with liver disease, defined as aspartate aminotransferase (AST) or alanine aminotransferase (ALT) greater than two times normal or total bilirubin > 2.5 mg/dl.
  - Patients with renal disease, defined as blood urea nitrogen (BUN) > 30 mg/dl or serum creatinine > 1.4 mg/dl.
  - Patients with hemoglobin < 10 g/dl.
  - Patients with a history of deep venous thrombosis, pulmonary embolus, or cerebrovascular accident.
  - Patients with known heart disease that is likely to be exacerbated by pregnancy.
  - Patients with a history of suspected cervical carcinoma, endometrial carcinoma, or breast carcinoma. A normal Papanicolaou test result will be required for women aged 21 years and over.
  - Patients with a current history of alcohol abuse. Alcohol abuse is defined as > 14 drinks/week or binge drinking.
  - Patients enrolled in other investigative studies that require medications, prescribe the study medications, limit intercourse, or otherwise prevent compliance with the protocol.
  - Patients who anticipate taking longer than a 1-month break during the protocol.
  - Patients with a suspected androgen-secreting adrenal or ovarian tumor.
  - Couples with previous sterilization procedures (vasectomy, tubal ligation) that have been reversed. The prior procedure might affect the study outcomes, and patients with both a reversed sterilization procedure and PCOS are rare enough that exclusion should not adversely affect recruitment.
  - Subjects who have undergone bariatric surgery in the recent past (< 12 months) and are in a period of acute weight loss or have been advised against pregnancy by their bariatric surgeon.
  - Patients with untreated or poorly controlled hypertension, defined as a systolic blood pressure ≥ 160 mmHg or a diastolic blood pressure ≥ 100 mmHg obtained in two measurements taken at least 60 min apart.
  - Patients with known congenital adrenal hyperplasia.

### Intervention

All participants will be informed of the benefits of regular physical exercise and will be instructed to have regular intercourse every 2 to 3 days. Women in both the personalized and fixed acupuncture groups will receive acupuncture treatment three times per week with a maximum of 48 treatment sessions over 16 weeks. Acupuncture treatment will be started between days 3 and 7 of the spontaneous menstrual cycle or between days 3 and 7 after withdrawal bleeding following progestin. They will be contacted by phone if they miss the scheduled appointment. If they miss appointments during treatment, missed appointments will be clearly documented in the record for later analysis. They will be treated for up to 16 weeks. If they become pregnant, the treatment they are receiving will be stopped. For the personalized and fixed acupuncture groups, credibility and expectancy questionnaires will be completed at the third acupuncture treatment and at the last acupuncture treatment.

#### Personalized acupuncture protocol

The rationale for the personalized acupuncture protocol is based on the zang-fu organ system, yin-yang theory, and clinical rules for PCOS acupoint selection [27]. Two sets of acupoints will be selected for the two types of deficiencies (Table 1). The basic acupoint prescription for patients in the personalized acupuncture group includes conception vessel (CV) 4, CV 6, and CV 12 and spleen (SP) 6 bilaterally; stomach (ST) 25 bilaterally; extra point of chest and abdomen 1 bilaterally; ST 40 bilaterally; and SP 9 bilaterally. Additional point ST 36 bilaterally and moxibustion as adjuvant therapy will be added for the yang-type deficiency of the spleen and kidney, whereas additional points kidney (K) 13 and liver (LR) 3 will be added for the yin-type deficiency of the liver and kidney. In addition, flexible modifications of two or three acupoints will be made according to patients’ specific symptoms.

**Table 1:** Personalized acupuncture protocol

Point	Stimulation	Location	Anatomy	Muscle innervation	Method
Spleen-yang and kidney-yang deficiency					
Sanyinjiao (bilateral) (SP6)	EA, (+)	3 cun proximal to the medial malleolus	M. flexor digitorum longus, tibialis posterior	L4–5, S1	Puncture perpendicularly 0.5–1.0 cun
Tianshu (bilateral) (ST25)	A	2 cun lateral to the midline at the level of the umbilicus	Muscle rectus abdominis	Th6–12	Puncture perpendicularly 0.7–1.2 cun
Zigong (bilateral) (EX-CA1)	A	1 cun cranial to the pubic bone and 3 cun lateral to the midline	M. obliquus externus abdominis, obliquus internus abdominis	Th12, L1	Puncture perpendicularly 0.8–1.2 cun
Guanyuan (CV4)	A	3 cun caudal to the umbilicus	Fibrous tissue, linea alba	Th12	Puncture perpendicularly 0.8–1.2 cun
Zhongwan (CV12)	A	4 cun superior to the umbilicus	Fibrous tissue, linea alba	Th8	Puncture perpendicularly 0.8–1.2 cun
Qihai (CV6)	A	1.5 cun caudal to the umbilicus	Fibrous tissue, linea alba	Th11	Puncture perpendicularly 0.8–1.2 cun
Fenglong (bilateral) (ST40)	De-qi 4 times, (−)	8 cun superior to lateral malleolus	M. extensor digitorum longus, peroneus M., M. gastrocnemius	L4–5, S1–2	Puncture perpendicularly 0.5–1.0 cun
Yinlingquan (bilateral) (SP9)	EA, (−)	Below the medial tibia chondyle	M. gastrocnemius	S1–2	Puncture perpendicularly 0.5–1.0 cun
Zusanli (bilateral)(ST36)	De-qi 4 times, (+)	On the anterior lateral side of the leg, 3 cun below Dubi (ST35), one finger width (middle finger) from the anterior crest of the tibia	M. tibialis anterior, extensor digitorum longus, M. tibialis posterior	L2–4	Puncture perpendicularly 0.5–1.0 cun
Liver-yin and kidney-yin deficiency					
Sanyinjiao (bilateral) (SP6)	EA, (+)	3 cun proximal to the medial malleolus	M. flexor digitorum longus, tibialis posterior	L4–5, S1	Puncture perpendicularly 0.5–1.0 cun
Tianshu (bilateral) (ST25)	A	3 cun caudal to the umbilicus	Fibrous tissue, linea alba	Th12	Puncture perpendicularly 0.7–1.2 cun
Zigong (bilateral) (EX-CA1)	A	1 cun cranial to the pubic bone and 3 cun lateral to the midline	M. obliquus externus abdominis, obliquus internus abdominis	Th12, L1	Puncture perpendicularly 0.8–1.2 cun
Guanyuan (CV4)	A	3 cun caudal to the umbilicus	Fibrous tissue, linea alba	Th12	Puncture perpendicularly 0.8–1.2 cun
Zhongwan (CV12)	A	4 cun superior to the umbilicus	Fibrous tissue, linea alba	Th8	Puncture perpendicularly 0.5–1.2 cun
Qihai (CV6)	A	1.5 cun caudal to the umbilicus	Fibrous tissue, linea alba	Th11	Puncture perpendicularly 0.8–1.2 cun
Fenglong (bilateral) (ST40)	Deqi 4 times, (−)	8 cun superior to the lateral malleolus	Mm. extensor digitorum longus, peroneus M., M. gastrocnemius	L4–5, S1–2	Puncture perpendicularly 0.5–1.0 cun
Yinlingquan (bilateral) (SP9)	EA, (−)	Below the medial tibia chondyle	M. gastrocnemius	S1–2	Puncture perpendicularly 0.5–1.0 cun
Taixi (bilateral) (K13)	Deqi 4 times, (+)	Behind the medial malleolus	Fibrous tissue	L3–4	Puncture perpendicularly 0.5–1.0 cun
Taichong (bilateral) (LR3)	Deqi 4 times, (−)	Between metatarsals I and II, just distal to the caput	M. interosseous dorsalis I	S2–3	Puncture perpendicularly 0.5–1.0 cun

*Abbreviations: A* acupuncture, *CV* conception vessel, *EA* electroacupuncture, *EX-CA* extra point of chest and abdomen, *GV* governor vessel, *K* kidney, *L* lumbar vertebra, *LI* large intestine, *LR* liver, *S* sacral vertebra, *ST* stomach, *SP* spleen, *Th* thoracic vertebra

+/− signifies that these points have been rotated bidirectionally for tonifying (+) or sedating (−)

The additional points ST 36, K 13, and LR 3 and all the basic points will be inserted with disposable needles (Huanqiu; Suzhou Acupuncture Goods Co., Suzhou, China) of size 0.30 mm × 25 mm and 0.30 mm × 40 mm. The depth and degree of puncture for each acupoint are described in Table 1, but these will be adjusted according to the body shape of the participants and will be shallower for thin women and deeper for overweight and obese women. The needles for abdominal acupoints will be inserted until resistance is felt without manual stimulation. After inserting the needles, the acupuncturist will rotate the needles to evoke needle sensation (de-qi). *De-qi* refers to a sensation of a dull aching, numbness, distention, or electrical tingling at the needling site that might radiate along the corresponding meridian and is indicative of effective needle placement. Once de-qi is achieved, further techniques might be used that aim to “influence” the de-qi, including using the thumb and forefinger to tonify or sedate qi. Tonifying is used to reinforce deficient qi, whereas sedating is used to clear excess qi. In tonifying, the needles are rotated more slowly and gently in a clockwise direction, whereas in sedating, the needles are turned faster and more forcefully in a counterclockwise direction. Furthermore, the points SP 6 to SP 9 bilaterally for both types will be connected to an electrical stimulator (SDZ-IIB; Huatuo, China) and stimulated with a low-frequency continuous wave at 2 Hz, and the intensity will be adjusted to produce local muscle contractions without pain or discomfort.

**Fig. 1:**
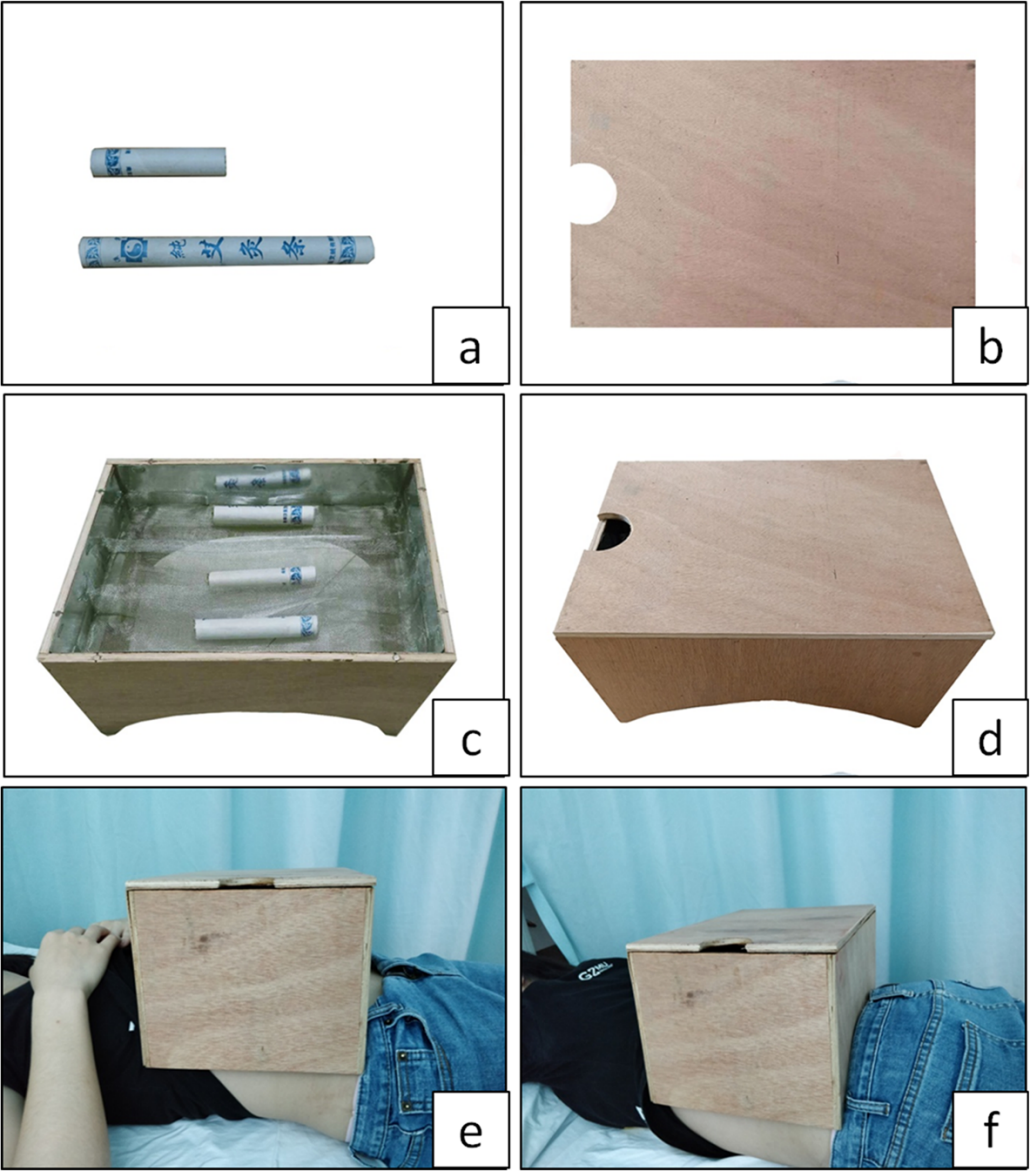
Moxibustion in the personalized acupuncture group. **a** 1.5 Moxa sticks used for a treatment session. **b** Safety lid of the moxa box. **c** Ignited moxa sticks placed in the moxa box. **d** The complete moxa box. **e** The moxa box placed on the abdomen of the patient from the midpoint of the sternal body xiphoid junction and umbilicus to the superior margin of the pubic symphysis. **f** The moxa box on the back of a patient from the 11th thoracic vertebra to the 4th sacral posterior foramen

Abdominal and back moxibustion will be used for the yang-type deficiency of the spleen and kidney (Fig. 1). Ignited 1.5 moxa sticks (18 mm × 200 mm; Hanyi, Nanyang, China) in a special moxa box (19.5 cm × 29.5 cm × 16.5 cm) will be used for each treatment session. The moxa box will be placed on the patient’s abdomen for 30 min at the same time as the acupuncture treatment. The abdominal moxa box will cover from the midpoint of the sternal body xiphoid junction and umbilicus to the superior margin of pubic symphysis. After 30 min, the moxa box will be placed on the patient’s back for another 30 min without acupuncture. The box will cover from the 11th thoracic vertebra to the 4th sacral posterior foramen. Moxibustion will be stopped at any time if the participants experience a dry mouth, canker sores, or sore throat. Women not treated with moxibustion will be given local radiation for the abdomen using a special electromagnetic spectrum therapy apparatus. Needles not connected to the electrical stimulator or not in abdominal acupoints will be manually stimulated to evoke de-qi every 10 min, and this stimulation will be repeated four times.

#### Fixed acupuncture protocol

The fixed acupuncture protocol is based on a previous study [10]. Two sets of acupuncture points will be alternated every other treatment (Table 2). The first set consists of CV 3, CV 6, ST 29 bilaterally, SP 6 bilaterally, SP 9 bilaterally, governor vessel (GV) 20, and large intestine (LI) 4 bilaterally. In total, 11 needles will be placed. The needles for abdominal acupoints should be inserted until resistance is felt without manual stimulation. Other needles will be manipulated manually by rotation to evoke de-qi. CV 3, CV 6 (midline), ST 29 bilaterally, SP 6 bilaterally, and SP 9 bilaterally will be connected to an electrical stimulator (SDZ-IIB; Huatuo, China). The second set consists of 13 needles: ST 25 bilaterally, ST 29 bilaterally, CV 3, CV 6, SP 6 bilaterally, LR 3 bilaterally, pericardium meridian (PC) 6 bilaterally, and GV 20. The following points will be connected to an electrical stimulator: ST 25 bilaterally, ST 29 bilaterally, SP 6 bilaterally, and LR 3 bilaterally. Electrical stimulation will be delivered with a low-frequency continuous wave of 2 Hz, and the intensity will be adjusted to produce local muscle contractions without pain or discomfort. An electromagnetic spectrum therapy apparatus will be used during the treatment. Needles not connected to the electrical stimulator and not in abdominal acupoints will be manually stimulated to evoke de-qi every 10 min, and the stimulation will be repeated four times.

**Table 2:** Fixed acupuncture protocol

Point	Stimulation	Location	Muscle	Muscle innervation	Method
Set 1					
Zhongji (CV3)	EA	4 cun caudal to the umbilicus	Fibrous tissue, linea alba	L1	Puncture perpendicularly 0.5–1.0 cun
Qihai (CV6)	EA	1.5 cun caudal to the umbilicus	Fibrous tissue, linea alba	Th11	Puncture perpendicularly 0.8–1.2 cun
Guilai (bilateral) (ST29)	EA	1 cun cranial to the pubic bone and 2 cun lateral to the midline	M. rectus abdominis	Th6–12	Puncture perpendicularly 0.7–1.2 cun
Sanyinjiao (bilateral) (SP6)	EA	3 cun proximal to the medial malleolus	M. flexor digitorum longus, tibialis posterior	L4–5, S1–2	Puncture perpendicularly 0.5–1.0 cun
Yinlingquan (bilateral) (SP9)	EA	Below the medial tibia chondyle	M. gastrocnemius	S1–2	Puncture perpendicularly 0.5–1.0 cun
Hegu (bilateral) (LI4)	De-qi 4 times	On the highest point at the musculi interosseus dorsalis	M. interosseus dorsalis I, lumbricalis II, adductor pollicis	C8, Th1	Puncture perpendicularly 0.5–1.0 cun
Baihui (GV20)	De-qi 4 times	On the top of the head	Aponeurosis epicranii	C2–3,N trigeminus	Puncture subcutaneously 0.3–0.5 cun
Set 2					
Tianshu (bilateral) (ST25)	EA	2 cun lateral to the midline at the level of the umbilicus	M. rectus abdominis	Th6–12	Puncture perpendicularly 0.7–1.2 cun
Guilai (bilateral) (ST29)	EA	1 cun cranial to the pubic bone and 2 cun lateral to the midline	M. rectus abdominis	Th6–12	Puncture perpendicularly 0.7–1.2 cun
Zhongji (CV3)	A	4 cun caudal to the umbilicus	Fibrous tissue, linea alba	L1	Puncture perpendicularly 0.5–1.0 cun
Qihai (CV6)	A	1.5 cun caudal to the umbilicus	Fibrous tissue, linea alba	Th11	Puncture perpendicularly 0.8–1.2 cun
Sanyinjiao (bilateral) (SP6)	EA	3 cun proximal to the medial malleolus	M. flexor digitorum longus, tibialis posterior	L4–5, S1–2	Puncture perpendicularly 0.8–1.2 cun
Taichong (bilateral) (LR3)	EA	Between metatarsal I and II, just distal to the caput	M. interosseus dorsalis I	S2–3	Puncture perpendicularly 0.3–0.5 cun
Neiguan (bilateral) (PC6)	De-qi 4 times	2 cun proximal to the processus styloideus radii, between the tendons of the palmaris longus and the flexor carpi radialis	M. flexor digitorum superficialis	C8, Th1	Puncture perpendicularly 0.5–0.8 cun
Baihui (GV20)	De-qi 4 times	On the top of the head	Aponeurosis epicranii	C2–3,N trigeminus	Puncture subcutaneously 0.3–0.5 cun

The two sets will be alternated for every other treatment

*Abbreviations: A* acupuncture, *C* cervical vertebra, *CV* conception vessel, *EA* electroacupuncture, *GV* governor vessel, *L* lumbar vertebra, *LI* large intestine, *LR* liver, *PC* pericardium, *S* sacral vertebra, *SP* spleen, *ST* stomach, *Th* thoracic vertebra

#### Letrozole

Women in the letrozole group will receive letrozole without acupuncture. One pill of letrozole (2.5 mg) (Femara; Novartis Pharmaceuticals, Basel, Switzerland) will be started between days 3 and 7 of the spontaneous menstrual cycle or between days 3 and 7 after a withdrawal bleeding following progestin. After the initial dose, one pill will be taken for the next 4 consecutive days (five pills total). If there is a response with ovulation (i.e., the serum progesterone level on day 21 or day 28 of the cycle is > 3 ng/ml), this dose will be maintained and will be given between days 3 and 7 of the next cycle. In those with no ovulatory response, day 28 of the cycle will be regarded as day 1 of the next cycle. For the new cycle, letrozole will be started on day 1, and the letrozole dose will be increased to 5 mg (two pills) per day for 5 consecutive days. If there is still no response, the dose will be increased to 7.5 mg (three pills) per day for 5 days in the next cycle. The maximum daily dose of letrozole will be 7.5 mg daily for 5 consecutive days.

#### Placebo letrozole

Women in this group will receive placebo letrozole with no acupuncture. Placebo letrozole will have the same appearance as letrozole (Dongyangguang Pharmaceutical Co., Ltd., Guangdong, China) and will be given according to the same schedule as letrozole.

### Randomization and patient allocation

The web-based Research Management database system (ResMan; www.medresman.org) will be used to allocate patients in a 1:1:1:1 ratio. The data coordinating center (DCC) statisticians will generate and validate the randomization scheme for the study before it is implemented in the ResMan database system. When a participant is enrolled, an investigator at the participating site will take out a sealed envelope and enter the identification number into the ResMan database system and will log in to a password-protected secured website designed by the DCC to determine the group allocation.

### Blinding

The acupuncture groups will be known only to the acupuncturists and the ResMan data manager. The letrozole and the placebo letrozole pills with the same appearance will be organized in a kit consisting of one cycle of pills for each participant, and one participant will at most take 45 pills over a total of 4 cycles. The sealed envelope with four cycles’ worth of pills will be labeled with an identification number mapped to the letrozole or placebo letrozole allocations, which will be known only to the ResMan staff. Participants and investigators will be blinded to the letrozole or placebo letrozole allocation. All outcome assessors and the statisticians performing the data analysis will be blinded to group allocation. Except for emergencies, unblinding of individual participants will not take place until all pregnant participants giving birth and the outcomes of the births have been reported to the DCC. In the event that emergency unblinding is needed, only the site principal investigators (PIs) will be able to unblind the participant to treatment by contacting the director of the DCC. The site PIs and DCC staff will receive notification from the central randomization service when emergency unblinding has occurred.

### Study-specific visits and procedures

**Fig. 2:**
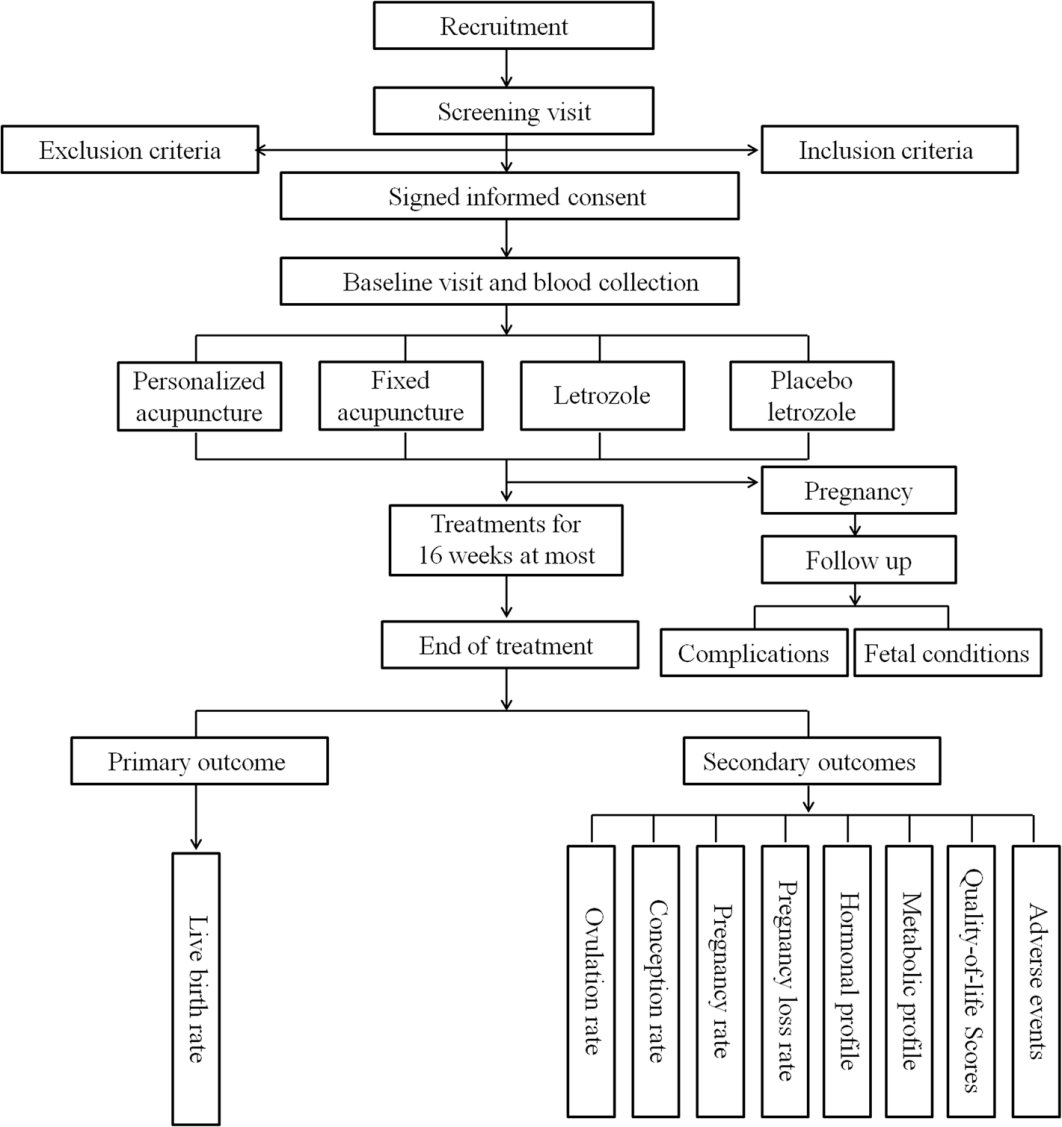
Study flowchart

Women will attend up to five different clinic visits, including the screening visit, the baseline visit, the treatment visits, the pregnancy visit, and the end-of-treatment visit (Fig. 2, Table 3). Adverse events (AEs) and concomitant medications will be recorded at every visit. Face-to-face adherence reminders will be given at each study visit.

**Table 3:** Overview of the study visits

	Screening visit	Baseline visit	Treatment visits (16 weeks)				End-of-treatment visit	Pregnancy visit
			Personalized acupuncture	Fixed acupuncture	Letrozole	Placebo letrozole		
Sign consent	×							
History	×						×	
Urine pregnancy test	×	×	×	×	×	×		
Physical examination	×						×	
Transvaginal ultrasound	×							×
Semen analysis	×							
Tubal patency test	×							
HPV or TCT	×							
Preconception counseling	×							
Fasting blood for laboratory tests	×						×	
Blood sample collection		×					×	
Serum progesterone assay	×		×	×	×	×		×
Serum HCG assay	×	×	×	×	×	×		×
Questionnaires		×					×	
Acupuncture treatment (three times weekly)			×	×				
Assess adverse events and concomitant medications			×	×	×	×	×	×
Pregnancy and neonatal records								×

Physical examination: weight, height, waist circumference, hip circumference, Ferriman-Gallwey/acne

Fasting blood for laboratory test: follicle-stimulating hormone, luteinizing hormone, sex hormone–binding globulin, total testosterone, free testosterone, estradiol, prolactin, fasting blood glucose, fasting insulin, C-peptide, glycated hemoglobin, cholesterol, triglycerides, high-density lipoprotein cholesterol, low-density lipoprotein cholesterol, complete blood count, thyroid profile, renal profile, and liver profile

Transvaginal ultrasound: endometrial thickness, ovarian volume, antral follicle count, and size of ovarian cysts or developing follicles

Preconception counseling: TORCH (toxoplasmosis, rubella, cytomegalovirus, and herpesvirus) and human immunodeficiency virus screening

Blood sample collection: serum for the central core laboratory

*Abbreviations: HCG* Human chorionic gonadotropin, *HPV* Human papillomavirus, *TCT* ThinPrep cytologic test

### Screening visit

Women will be screened in the morning after an overnight 12-h fast. Detailed information about the study design will be given.

#### Physical examination

Complete physical examinations will be performed, including measurement of height, weight, hip, and waist and vital signs. Height and weight will be recorded to the nearest 1 cm and 0.1 kg, respectively. Waist and hip circumference will be recorded to the nearest 1 cm. Hirsutism will be assessment by FG score, and acne will be assessed by standard acne lesion counts.

#### Transvaginal ultrasound of ovaries

Ovary size in three dimensions, the size of the largest ovarian follicle/cyst, the size of every follicle with a mean diameter > 10 mm, and the total antral follicle count (small follicles with a mean diameter of 2–9 mm) of both ovaries will be obtained through transvaginal ultrasound.

#### Pregnancy test

A urine and serum pregnancy test will be performed to exclude pregnancy.

#### Laboratory tests

Blood samples will be collected for the following measurements: total T, sex hormone–binding globulin (SHBG), FSH, LH, progesterone (P), estradiol (E2), PRL, glucose and insulin concentrations, HbA1C, C-peptide, and lipid profile (cholesterol, triglycerides, high-density lipoprotein cholesterol [HDL-C], and low-density lipoprotein cholesterol [LDL-C]), liver profile (ALT, AST, and total bilirubin), renal profile (BUN and creatinine), thyroid profile (TSH, T3, and free T4), complete blood count, and human papillomavirus test or ThinPrep cytologic test. All of these parameters will be measured in the local laboratories at each study site. The free androgen index will be calculated using measurements of total T and SHBG as previously described [5].

#### Preconception counseling

TORCH screening (toxoplasma, rubella virus, cytomegalovirus, herpesvirus), human immunodeficiency virus screening, and folic acid prescription will be included.

#### Progestin withdrawal

Women without evidence of ovulation on baseline screening will be provided progestin to induce withdrawal bleeding, with instructions to begin medication once eligibility has been determined. All participants will sign the consent form after the comprehensive counseling.

### Baseline visit

The baseline visit will take place around days 1–7 of a spontaneous menstruation cycle or after a withdrawal bleeding if a woman fulfills the inclusion criteria and has signed the consent form. Fasting blood samples will be obtained from the patients and will be shipped to the core laboratory for analysis and stored for a minimum of 5 years and may be further used in future studies, which is stated in the informed consent form (Supplementary file 2). Moreover, a urine or serum pregnancy test will be performed at this visit.

#### Questionnaires

Women will complete the following questionnaires: the 36-item Short Form Health Survey (SF-36) of health-related quality of life (QOL), the Polycystic Ovary Syndrome Quality of Life Questionnaire (PCOS-QOL), the Chinese Quality of Life (ChQOL), the Pittsburgh Sleep Quality Index (PSQI), the Zung Self-Rating Anxiety Scale (SAS), and the Zung Self-Rating Depression Scale (SDS).

The symptoms of PCOS are mainly differentiated in TCM as a liver-kidney yin-type deficiency or a spleen-kidney yang-type deficiency. The TCM syndrome diagnosis will be made by the PI and acupuncturist in each participating site according to a standard questionnaire during the baseline visit and the end-of-treatment visit. If the diagnosis differs between the PI and acupuncturist, they will discuss this until reaching an agreement. Syndrome differentiation in TCM entails the comprehensive analysis of clinical information gained by the four main diagnostic TCM procedures: observation, listening, questioning, and pulse analysis. All baseline measures will be repeated in all subjects at the end-of-treatment visit.

### Treatment visits

The women will be instructed to engage in intercourse once every 2 or 3 days. A serum progesterone measurement will be taken in the local laboratory on day 21 of the menstruation cycle. An elevated progesterone level > 3 ng/ml will be considered to indicate evidence of ovulation. If there is no ovulation, the serum progesterone level will be checked every week until ovulation. If ovulation occurs, a serum pregnancy test will be performed 1 week later. For the duration of the treatment, urine LH or human chorionic gonadotropin (HCG) levels can be measured, depending on the judgment of the PI, in order to obtain timely information on ovulation or pregnancy and thus to instruct the participant to continue engaging in intercourse or to stop the intervention. We will determine whether the women have an ovulation response based on the serum progesterone level, and there will be three possible scenarios—ovulation, delayed ovulation, or no ovulation—as follows:

1. *Ovulation*: Serum progesterone level on day 21 of the cycle > 3 ng/ml. A serum pregnancy test will be performed 1 week later to exclude pregnancy. In the letrozole and placebo letrozole groups, letrozole or placebo letrozole will be started between days 3 and 7 of the new cycle, and the letrozole or placebo letrozole dose will be maintained at the same level as the previous cycle.
2. *Delayed ovulation*: Serum progesterone level on day 21 of the cycle < 3 ng/ml. In this case, we will wait 1 week and then check the serum progesterone level again. If the second measurement shows that ovulation occurred (serum progesterone > 3 ng/ml), a serum pregnancy test will be performed 1 week later to exclude pregnancy. In the letrozole and placebo letrozole groups, letrozole or placebo letrozole will be started between days 3 and 7 of the new cycle, and the letrozole or placebo letrozole dose will be maintained.
3. *No ovulation*: Negative serum progesterone on day 21 and day 28. In the letrozole and placebo letrozole groups, women will take letrozole or placebo letrozole on day 28 of the cycle, and then one more tablet per day will be given for 5 consecutive days until the maximum daily dose of three tablets per day. Day 28 will be defined as day 1 of the next cycle, and a serum progesterone measurement will be made every week until ovulation.

Every menstruation will be recorded, including the date, amount, and duration of menstruation.

### End-of-treatment visit

The end-of-treatment visit will be performed when the women conceive or finish four cycles of treatment. Baseline measures will be repeated in all subjects, including vital signs, height, weight, hip, and waist measurements; hirsutism and acne assessments; sex hormone steroids (FSH, LH, and total T for women who are not pregnant and β-HCG, P, E2, and total T for women who are pregnant); SHBG; metabolic profile (glucose and insulin concentrations, HbA1C, C-peptide, cholesterol, triglycerides, HDL-C, and LDL-C); liver profile (AST, ALT, and total bilirubin); and renal profile (BUN and creatinine). Subjects will return menstrual and intercourse journal logs, and their AEs and concomitant medications will be recorded. Fasting blood samples will be obtained, and TCM syndrome diagnosis and the SF-36, PCOS-QOL, ChQOL, PSQI, Zung SAS, and Zung SDS questionnaires will be repeated.

### Pregnancy visit (only with conception)

Serum progesterone and β-HCG levels will be determined at local sites to document ovulation and pregnancy. A serum β-HCG > 10 IU/L will indicate pregnancy, and in pregnant women, the serum β-HCG level will continue to be checked on a weekly basis. Pelvic ultrasound will be performed to determine the location and number of fetuses when the serum β-HCG is 2000–4000 IU/L. Pelvic ultrasound will then be performed every 2 weeks until viability of the pregnancy is determined by visualization of fetal heart motion.

Women who conceive will be referred to their prior or referring doctors for antenatal care at around 10–12 weeks of gestational age. Subsequent arrangements will be performed at weeks 18–24, 32, and 36 or upon the prescription of the obstetrician. In the present trial, pregnant women who present with threatened miscarriage or who are considered to have a significant risk for miscarriage can be given oral Duphaston (10 mg three times per day; Solvay Pharmaceuticals B.V., Veenendaal, the Netherlands) until 12 weeks of gestation or until 1 week after the vaginal bleeding stops. This information will be documented in the study records. No other medications, including HCG, herbal medications, or acupuncture, will be given.

### Follow-up of pregnancies

Women who conceive will be referred to their prior or referring doctors for antenatal care at around 10–12 weeks of gestational age, and they will be seen in follow-up until delivery or termination of gestation. All pregnancies (including multiples) will be followed to monitor weight, glucose tolerance, blood pressure, and fetal growth and to determine the pregnancy outcomes. Glucose tolerance will be measured by oral glucose tolerance test in all pregnant women at 24–28 weeks of pregnancy. Women will be instructed to inform the study personnel of the outcomes of the pregnancy, and we will obtain delivery records from the obstetricians to determine the birth weight, length of gestation, and any prenatal complications in the mother or neonatal complications in the infant. Phone contacts will be initiated if the pregnant woman has not contacted the study personnel within 6 weeks of the original estimated date of childbirth.

We will collect pregnancy outcome data and track the outcomes of all women who have a positive serum pregnancy screen during the course of this study. We will record biochemical pregnancies (defined as positive serum pregnancy screens without ultrasonically detected pregnancies), ectopic pregnancies, and all intrauterine pregnancy losses both before and after 20 weeks, including miscarriages, abortions, fetal deaths, and stillbirths.

### Outcomes

#### Primary outcome

The primary outcome is the live birth rate, defined as delivery of any viable infant after 28 weeks of gestation.

#### Secondary outcomes

The secondary outcomes include ovulation rate, conception rate, pregnancy rate, pregnancy loss rate, changes in hormonal/metabolic parameters, changes in QOL scores, and changes in AEs.

#### Ovulation rate

The ovulation rate per participant and per cycle will be analyzed. Ovulation will be defined when the serum progesterone level on day 21 or day 28 of the cycle is > 3 ng/ml. The ovulation rate per participant will be calculated as the proportion of women who ovulated at least once during the treatments among the total women in the primary analysis. The ovulation rate per cycle will be calculated as the proportion of cycles in which ovulation occurs among all tested cycles in the primary analysis.

#### Conception rate

Conception will be defined as a positive serum β-HCG measurement. Thus, the cumulative conception rate will be the proportion of women who conceive during the four treatment cycles among all women in the primary analysis.

#### Pregnancy rate

Pregnancy will be defined as an intrauterine pregnancy with fetal heart motion as determined by transvaginal ultrasound. The cumulative pregnancy rate will be the proportion of pregnant women among the total participants in the primary analysis.

#### Pregnancy loss rate

Pregnancy loss will be defined as pregnancy loss occurring from conception to 27 completed weeks of gestational age in this trial, including pregnancy loss in the first trimester, pregnancy loss in the second or third trimester, biochemical pregnancy loss, and ectopic pregnancy. The pregnancy loss rate will be the proportion of women who abort among all participants who conceive.

#### Changes in hormonal/metabolic parameters

Hormonal/metabolic profiles will be determined at the baseline visit and the end-of-treatment visit. The mean values of changes in hormonal/metabolic parameters before and after treatments will be used in the primary analysis.

#### Changes in QOL scores

QOL scores will be assessed by the SF-36, PCOS-QOL, ChQOL, PSQI, Zung SAS, and Zung SDS questionnaires. The participants will complete the questionnaires at the baseline visit and again at the end-of-treatment visit. Higher scores on the SF-36, PCOS-QOL, and ChQOL questionnaires indicate better function, whereas higher scores on the PSQI indicate poorer sleep quality, and higher scores on the Zung SAS/SDS indicate worse anxiety/depression. The mean changes in QOL scores of each group will be used in the analysis.

#### Adverse events

All AEs will be categorized, and the percentage of patients experiencing AEs and serious adverse events (SAEs) during the treatment period and follow-up period will be documented and reported. For fetuses, SAEs and AEs will be reported as those occurring after 20 weeks of gestation through birth, and for newborns, SAEs and AEs will be reported as those occurring within 28 days after birth. SAEs will include events that are fatal or immediately life-threatening, severely or permanently disabling, or that require hospitalization; overdoses (intentional or accidental); congenital anomalies; pregnancy loss after 12 weeks of gestation; neonatal death up to 6 weeks after delivery; or any event deemed to be serious by the site PIs. The Data and Safety Monitoring Board (DSMB) will review AEs on a quarterly basis and will review SAEs immediately.

### Sample size calculations

In previous studies, the overall live birth rate for infertile women with PCOS who received letrozole for 6 months was 36.3% [28], whereas for women who received fixed acupuncture twice per week with a maximum of 32 acupuncture treatments, the overall live birth rate was 13.9% [10]. Thus, we hypothesize that the live birth rate is 35% for four cycles of letrozole treatments and 15% for fixed acupuncture three times per week. We have chosen 10% as the minimal clinically detectable difference that is likely to change clinical practice. Assuming a 35% live birth rate with letrozole, a 25% live birth rate with personalized acupuncture, a 15% live birth rate with fixed acupuncture, a 5% live birth rate with placebo letrozole, and an 80% power at a significance level of 0.05, we calculated that 250 subjects per treatment group were required. The sample size was increased from 250 to 275 per group considering a 10% dropout rate, and thus 1100 women in total will be enrolled.

### Data analysis

The primary analysis will use an intention-to-treat approach to examine differences in the live birth rate among the four treatment groups. Primary efficacy analysis will be performed by comparing the treatment groups with respect to the primary outcome of live birth using the Pearson chi-square test. For the secondary supportive analysis, we will fit a logistic regression model to compare the treatment arms with respect to the primary outcome of live birth, adjusting for other factors such as randomization stratification of study site and prior exposure to study medications. Comparisons of variables such as live birth rate, ovulation rate, conception rate, pregnancy rate, multiple pregnancy rate, and miscarriage rate will include relative risk and 95% confidence intervals in addition to the chi-square test. Changes in laboratory values will be assessed by analysis of covariance (ANCOVA).

The analysis of secondary (supplemental) outcomes measured over time will entail the application of statistical methods that have been developed for correlated data because repeated observations will be made over time for each individual. For secondary outcomes such as hormone levels, a linear mixed-effect model will be fit where the main independent variables will be treatment group, time, and their interaction as well as the designed randomization stratification factors as covariates. Logistic regression models will be used in secondary analyses to evaluate the predictive value of treatment arm, clinical site, body mass index, and other explanatory variables on binary outcomes (e.g., live birth, abortion). Cox proportional hazards models and the Kaplan-Meier method will be used to compare time to pregnancy in the treatment groups. Effects on QOL, as determined by the change from baseline physical and mental component scores, will be analyzed by univariate and multivariate ANCOVA. The adequacy of blinding will be tested by Fisher’s exact test comparing true group assignments with participant guesses, including “don’t know.” In order to evaluate acupuncture as a unique therapy, the combination of the personalized acupuncture group and fixed acupuncture group will also be compared with the combination of the letrozole and placebo letrozole groups for the analysis of primary outcome. Differences in AE rates will be analyzed in a negative binomial generalized linear model. A *P* value < 0.05 with a two-tailed test will be considered significant. All statistical analyses will be performed using IBM SPSS Statistics version 21.0 software (IBM Corp., Armonk, NY, USA).

### Imputation procedure for missing data

We will report the reasons for withdrawal for the different randomization groups and will compare the reasons qualitatively. The effect that any missing data might have on the results will be assessed via sensitivity analysis of augmented data sets. Dropouts (essentially participants who withdraw consent for continued follow-up) will be included in the analysis using modern imputation methods for missing data.

### Quality assurance

To ensure that treatments are of a high standard and delivered in accordance with the trial protocol, all acupuncturists will have a TCM certificate and at least 1 year of experience, will take a study-specific theoretical course, and will undergo study-specific practical training. Three training sessions lasting 1–3 days each will take place before the beginning of the study, and then a single 4-h training session will be given 1 month after the beginning of the study. The acupuncturists are graduate students in TCM and have received general acupuncture training during their studies. All acupuncturists have to pass the theoretical and practical tests.

### Study governance and management

The study will be led by a steering committee consisting of an independently selected chair, site PIs, and other investigators. Each of these individuals will have one vote, and decisions will be reached by majority consensus and a formal vote. The steering committee will meet face to face four times per year and monthly via phone conferences and will communicate through email on a regular basis. Additionally, the study will have subcommittees, including a protocol committee, a recruitment committee, and a publication committee.

The independent DSMB will hold regular conference calls to review the protocol with respect to ethical and safety standards, to monitor the safety of the trials, to monitor the integrity of the data with respect to the original study design, and to provide advice on study conduct. The DSMB will also have the power to terminate the study for a variety of reasons, including poor recruitment, AEs caused by the study medications, or a clear trend in live births in one of the blinded treatment arms exceeding our expectations.

### Data access, management, and quality control

Both the case report form (CRF) and a web-based electronic database will be used to manage individual participant data. Quality control of the data will be handled at three different levels. First, the investigators will be required to ensure the accuracy of the data when they input the data from the CRF. The second level will include data monitoring and validation that will be carried out by the DCC staff. The third level will be the site visits, during which the data in ResMan will be compared with the source documents. Identified errors will be resolved by ResMan and the clinical sites.

The ResMan database will be used as a double-input database. The ResMan staff will oversee the intrastudy data-sharing process. All PIs will be given access to the cleaned data sets. Project data sets will be housed on the ResMan database system for the duration of the study, and all data sets will be password-protected. The PIs will have direct access to their own site’s data sets as well as access to other sites’ data by request. To ensure confidentiality, data dispersed to project team members will be blinded to any identifying participant information.

### Ethics and dissemination

All women and their husbands will be asked to sign a consent form prior to joining the study, and they will be made fully aware that they are free to withdraw from the study at any time. The results of this trial will be disseminated in peer-reviewed journals and presented at international meetings.

## Discussion

To the best of our knowledge, no previous study has investigated whether personalized acupuncture has the potential to increase the live birth rate in infertile women with PCOS. Our study is thus the first multicenter randomized controlled trial to compare the effects of personalized or fixed acupuncture with letrozole or placebo letrozole on live birth in infertile women with PCOS. The hypothesis is that letrozole is more effective than personalized acupuncture, that personalized acupuncture is more effective than fixed acupuncture, and that fixed acupuncture is more effective than placebo letrozole. Moreover, we hypothesize that personalized acupuncture is more likely than letrozole to reduce miscarriage rate and the risk of pregnancy complications.

Considering the treatment principle based on syndrome/pattern differentiation in TCM, the kidney, liver, and spleen are the top three zang-fu organs associated with the development of PCOS, and yin or yang deficiency is the essential cause, whereas pathological products such as phlegm are superficial [29]. Therefore, the therapeutic proposal should reinforce liver-kidney-yin deficiency, promote spleen-kidney-yang circulation, and resolve phlegm. We will divide the individual groups into two types according the theory of TCM as “liver-yin and kidney-yin deficiency” and “spleen-yang and kidney-yang deficiency.” This method is similar to that of actual clinical practice and can be used in future studies. The fixed acupuncture regimen is controversial based on the TCM theory of acupuncture.

## Trial status

This trial was approved on March 9, 2018. The recruitment began on August 13, 2018, and is expected to be completed by August 13, 2021. The trial procedures are expected to be completed by the end of May 2022, when considering 10-month pregnancies and the follow-up time for collecting information regarding live births. This is version 3.0 of the protocol, dated May 12, 2019.

## Supplementary information

**Additional file 1 **: **Supplementary file 1**. SPIRIT 2013 checklist: recommended items to address in a clinical trial protocol and related documents.

**Additional file 2 **: **Supplementary file 2.** Patient information and consent.

## Data Availability

The supplementary data, except for private information, will be stored on a public website (e.g., Dryad, https://datadryad.org/stash).

## Abbreviations

AE: Adverse event
ALT: Alanine aminotransferase
ANCOVA: Analysis of covariance
AST: Aspartate aminotransferase
BUN: Blood urea nitrogen
ChQOL: Chinese Quality of Life instrument
CONSORT: Consolidated Standards of Reporting Trials
CRF: Case report form
CV: Conception vessel
DCC: Data coordinating center
DSMB: Data and safety monitoring board
E2: Estradiol
EX-CA: Extra point of chest and abdomen
FG: Ferriman-Gallwey
FSH: Follicle-stimulating hormone
GV: Governor vessel
HbA1c: Glycated hemoglobin
HCG: Human chorionic gonadotropin
HDL-C: High-density lipoprotein cholesterol
HPV: Human papillomavirus
K: Kidney
LDL-C: Low-density lipoprotein cholesterol
LH: Luteinizing hormone
LI: Large intestine
LR: Liver
P: Progesterone
PC: Pericardium meridian
PCOS: Polycystic ovary syndrome
PCOS-QOL: Polycystic Ovary Syndrome Quality of Life Questionnaire
PI: Principal investigator
PRL: Prolactin
PSQI: Pittsburgh Sleep Quality Index
QOL: Quality of life
RCT: Randomized controlled trial
ResMan: Research Management
SAE: Serious adverse event
SAS: Zung Self-Rating Anxiety Scale
SDS: Zung Self-Rating Depression Scale
SF-36: 36-Item Short Form Health Survey
SHBG: Sex hormone–binding globulin
SP: Spleen
SPIRIT: Standard Protocol Items: Recommendations for Interventional Trials
ST: Stomach
STRICTA: Standards for Reporting Interventions in Clinical Trials of Acupuncture
T: Testosterone
TCM: Traditional Chinese medicine
TCT: ThinPrep cytologic test
TORCH: Toxoplasma, rubella virus, cytomegalovirus, herpesvirus
TSH: Thyroid-stimulating hormone
