# Supplementary material for: A multicenter randomized trial of personalized acupuncture, fixed acupuncture, letrozole, and placebo letrozole on live birth in infertile women with polycystic ovary syndrome
Source: Trials. 2020 Mar 4;21:239. doi: 10.1186/s13063-020-4154-1 (PMC7057514; doi:10.1186/s13063-020-4154-1)
Supplement: Supplementary file 2 — Additional file 2 : Supplementary file 2. Patient information and consent. [file 13063_2020_4154_MOESM2_ESM.docx]

Patient information and consent

**A multicenter randomized trial of** **personalized acupuncture,** **fixed acupuncture, letrozole and placebo on live birth for infertility in women with polycystic ovary syndrome**

We sincerely invite you to participate in the aboved-named study because you have a condition known as Polycystic Ovary Syndrome (PCOS). This form gives you information about this study. Please ask questions about anything that is unclear to you. Discuss it with your family and friends and take your time to make up your mind.

**Why is this study being done?**Acupuncture has been used in China for more than 3000 years and is a core part of traditional Chinese medicine In recent years, the use of acupuncture as an adjuvant treatment to the management of various clinical conditions has gained increasing popularity worldwide. The underlying mechanisms of the benefits of acupuncture may be via modulation of endogenous regulatory systems, including the sympathetic nervous system, the endocrine system and the neuroendocrine system.

PCOS is a common endocrine disorder in women of reproductive age. Letrozole is the first-line therapy for infertility in women with PCOS but can not reduce miscarriage rate and the risk of pregnancy complications. Acupuncture has been used as an alternative therapy to induce ovulation in women with PCOS with few side effects. In the majority of studies on acupuncture, fixed acupuncture is used. In traditional Chinese medicine, acupuncture should be personalized according symptoms and signs of each patient in differential symptom diagnosis.

Therefore, we aim to investigate the efficacy of personalized acupuncture based on the TCM theory, fixed acupuncture, letrozole and placebo letrozole on the live birth rate of infertility women with PCOS.

**Aim of the study**The objectives of the present trial are to test the following four hypotheses for infertility in women with PCOS:

1. Letrozole is more likely to induce ovulation and results in live birth than personalized acupuncture.
2. Personalized acupuncture is more likely to induce ovulation and results in live birth than fixed acupuncture.
3. Fixed acupuncture is more likely to induce ovulation and results in live birth than placebo letrozole.
4. Personalized acupuncture is more likely to reduce miscarriage rate and the risk of pregnancy complications compared with letrozole, fixed acupuncture and placebo letrozole.

**You will randomly be allocated to one of four groups:**A) Personalized acupuncture
B) Fixed acupuncture
C) Letrozole
D) Placebo letrozole

**Who should be in this study?
You will be included in this study if you have the following:**

1. Age of women between 20 and 40 years.
2. Confirmed diagnosis of PCOS according to the Rotterdam criteria: Oligo-, amenorrhea (less than 8 cycles per year) and one of the following two criteria; clinical or biochemical hyperandrogenism and/or polycystic ovarian morphology.
3. At least one patent tube shown by hysterosalpingogram or diagnostic laparoscopy within 3 years if the patient does not have a history of abortion or pelvic operation. If the patient has a history of pregnancy and no history of pelvic operation within the past 5 years, she is not required to undergo a tubal patency test.
4. Sperm concentration ⩾15 × 10^6^/mL and total motility ⩾40% or total motile sperm count ⩾9 million in the semen analysis of the husband.
5. The couple agree to have regular intercourse i.e. 2-3 times per week during the study period.

**You will not be included in this study if you have the following:**

1.Exclusion of other endocrine disorders:

1) patients with hyperprolactinemia ( defined as two prolactin levels at least one week apart ⩾25 ng/mL)

2) Patients with FSH levels >15 mIU/mL. A normal level within the last year is adequate for entry.

3) Patients with uncorrected thyroid disease (defined as TSH <0.2 mIU/mL or >5.5 mIU/mL). A normal level within the last year is adequate for entry.

4) Patients diagnosed with Type I or Type II diabetes who are poorly controlled (defined as HbA1c level > 7.0%), or patients receiving antidiabetic medications such as metformin, insulin, thiazolidinediones, acarbose, or sulfonylureas.

5) Patients with suspected Cushing's syndrome.

2. Use of hormonal or other medication including Chinese herbal prescriptions in the past 3 months.

3. Pregnancy within the past 6 weeks.

4. Within 6 weeks postabortion or postpartum.

5. Breastfeeding within the last 6 months.

6. Not willing to give written consent to the study.

**What will I be asked to do?**If you fulfil the inclusion criteria and accept the study design, baseline measurements including measurements of body weight, height, waist circumference, hirsutism, fasting blood will be drawn for analyses of specific hormones. Also, TORCH (Toxoplasmosis, Rubella, Cytomegalo Virus, Herpes Virus) and HIV screening should be performed before you are recruited and your HIV status should be negative. On day 3 to day 7 after a spontaneous menstrual bleeding or a withdrawal bleeding following progestin, you will receive a call from the study coordinator regarding the result of the serum or urine pregnancy test taken. If the test is negative, then you will start with acupuncture treatment (30-60 minutes/per treatment session) three times a week during 16 weeks, up to a total of 48 treatments, or receive letrozole/placebo letrozole orally.

During the entire study period, you are asked to have regular intercourse, every 2-3 day. Intercourse frequency should be reported each month. A serum progesterone assay, which gives information if you have ovulated, will be performed weekly in the local laboratory from day 21 of the cycle until ovulation, and HCG assay which indicates if you are pregnant will be performed one week after ovulation. After the last treatment, all baseline measurement should be repeated.

**How long will I be in the study?**The treatments will last for 16 weeks. You will be in the study for about 20 weeks including screening and waiting for withdraw bleeding.

**How many women will participate in the study?**We plan to recruit 1100 women.

**What benefit can I expect?**You will not be charged for any laboratory tests and ultrasound examinations related to this study after you sign the consent form. You will receive acupuncture or medication treatments for free. The potential benefit to you is that the treatment you receive may prove to be more effective than the other study drug treatment or other available treatments, and you may become pregnant, although this cannot be guaranteed. With the results of your hormone testing, you may have a greater understanding of your health status.

**For what will you need to pay?**You are expected to have standard tests results before you can be recruited in the study. Those standard tests are uniform across our study sites and will be presented and explained to you before you agree to participate in this study. Similarly and hopefully, if a pregnancy results from your treatment, you will be responsible for paying for the care of pregnancy and delivery.

**What adverse (bad) effects may happen to me by participating in the study?**The major risks to the subjects are side effects from acupuncture and letrozole. Acupuncture is a safe procedure with few side-effects. It may occur during or after the treatment as pricking pain when penetrating the skin, small bleeding when needles are withdrawn, bruising, nausea, dizziness and fainting which all are short-lasting and mild. Symptoms like nausea, dizziness and fainting are almost completely eliminated by treating in supine position. The acupuncture needles will be used only once to avoid the risk of cross infection.

After taking letrozole, there are some occasional discomforts such as fatigue, dizziness, nausea, hot flashes, arthritis pain in your joints, back pain, increased cholesterol levels, formation of ovarian cysts, multiple pregnancies.

Please let us know the adverse reactions that have occurred, we will give you appropriate guidance.

Side effects are usually temporary and manageable. However, it is possible they could be more serious. In addition to the risks described above, there may be unknown risks we cannot predict while participating in this research. It is important that you notify your study coordinator if you experience any of these symptoms listed above and keep accurate documentation of these side effects on your daily journal logs.

**Protection of human subjects section**You will be seen on a weekly basis and we will record at this time any unusual side effects. We will be obtaining regular pregnancy tests in both urine and serum and will stop both medicine and acupuncture treatments as soon as you have a positive pregnancy test. You will be followed through study, after a positive pregnancy test, to confirm the location and viability of the pregnancy before being referred to your doctors taking care of pregnancy. It is possible that your pregnancy may be nonviable or a pregnancy is detected in your fallopian tube and will require further medical or surgical treatment. You will have the option to follow up with your own physician or be followed with us and with your permission, notify your physician regarding abnormalities.

We will be recording any adverse events, including serious ones. They will be reviewed on a quarterly basis by the Data Safety Monitor Board, and any serious adverse events will be immediately reviewed. Although there is always the possibility of private information being seen by others, we will take security measures to protect your data and confidential.

**Can I get compensation in the study?**

You will not receive any compensation for being in this research study. Every effort to prevent injury as a result of your participation will be taken. It is possible, however, that you could develop complications or injuries as a result of participating in this research study. In the event of injury resulting from this research, you will get the medical treatment for free without any additional financial compensation.

**Can I refuse to be in the study?**Your participation in this study is voluntary. You can choose not to take part in the study, or you can drop out at any time. You will not lose any benefits to which you are otherwise entitled. If you quit the study, you will receive the standard treatment as other patients at our Department.

**Blood samples collection and repository for** **future use**

As part of this study, we are obtaining blood from you. If you agree, the researchers would like to store your blood samples so that your blood can be studied in the future after this study is over. These future studies may provide additional information that will be helpful in understanding polycystic ovary syndrome, but it is unlikely that these studies will have a direct benefit to you. The results of these tests will not have an effect on your care. Neither your doctor nor you will receive results of these future research tests, nor will the results be put in your health record.

Your blood samples will be collected at the baseline visit and end of treatment visit, and stored on site for up to 3 months, then shipped to our core laboratory center. You are free to change your mind regarding the use of your sample. You should contact the site investigators of this study and let him/her know you wish to withdraw your permission for your blood samples to be used for testing. Your blood samples will be destroyed at that time of withdrawal. You understand that you will not have access to the sample once it is sent to the laboratory. If you consent to the collection of your blood samples, it will be kept for five years.

**Confidentiality and privacy**The investigators always maintain a strict privacy policy. All correspondence to the department is held confidentially; furthermore, at no time will your personal and/or identifying information be shared outside of our organization, for any reason.

Subjects have the rights of access to personal data and known study results, if any when needed, you enjoy or may enjoy rights for the protection of the confidentiality of your personal data, such as those regarding the collection, custody, retention, management, control, use (including analysis or comparison). By signing and dating this Consent Form, you understand and agree to all of the abovementioned conditions and rights. For any query, you should consult the researchers who in charge of this study so that your full awareness and understanding of the significance of compliance with the law governing privacy data is assured.

For any questions, you can consult the privacy director about privacy data or his office (020-83062452) regarding appropriate monitoring or oversight of your personal data protection. So your full understanding of the importance of complying with laws to manage privacy data is warranted.

For questions about the study or reporting of adverse events, please call your physician at telephone.

- I agree to save a blood sample for future studies.
- I have read and understood this consent form. All my questions have been answered. I volunteer to take part in this study.


  _____________________________ ________________________
  Subject's signature Date
  _____________________________ ________________________

Site Investigator’s signature Date
_____________________________ ________________________

Witness’s signature Date
